# My Second Thoughts About Face and Neck Lifting: A Video Interview With Dr Mario Pelle-Ceravolo

**DOI:** 10.1093/asjof/ojad104

**Published:** 2023-11-29

**Authors:** Mario Pelle-Ceravolo, Lorne King Rosenfield

Welcome to another edition of Second Thoughts on First Thoughts. Today, we are privileged to have a truly master surgeon, Dr Mario Pelle-Ceravolo, from Rome, Italy, talk to us about his experience first, delving in and then out … and finally back into the neck as he navigated along his own facelift learning curve! Pelle proves the profound efficacy of constantly tweaking one's technique: in this case, his evolution to a more curated strategy of more or less direct neck work depending upon the patient's physiognomy. Thanks for watching! (Video).

